# A Systematic Method for the Identification of Oligosaccharide Constituents in *Polygonatum cyrtonema* Hua Using UHPLC-Q-Exactive Orbitrap Mass Spectrometry

**DOI:** 10.3390/molecules30071433

**Published:** 2025-03-24

**Authors:** Suyu Yang, Bowen Gao, Qingrui Yang, Yanghui Huo, Kailin Li, Liangyin Shu, Lingxuan Fan, Yiliang Liu, Huanting Li, Wei Cai

**Affiliations:** 1 School of Pharmacy, Baotou Medical College, Baotou 014040, China; 17332325609@163.com (S.Y.); gaobw001@163.com (B.G.); qingruiyangjj@163.com (Q.Y.); hyanghui818@163.com (Y.H.); flxuan930@163.com (L.F.); 15304710658@163.com (Y.L.); 2 School of Pharmaceutical Sciences, Hunan University of Medicine, Huaihua 418000, China; lkl20182022@163.com (K.L.); shuliangyin@foxmail.com (L.S.); 3 School of Pharmacy, Shandong Second Medical University, Weifang 261053, China

**Keywords:** *Polygonatum cyrtonema*, characterization, fructo-oligosaccharides, arabino-oligosaccharides

## Abstract

A *Polygonatum cyrtonema* Hua (PCH) is a common medicinal and edible plant whose rhizomes are widely used for the treatment and prevention of various diseases. Previous studies have revealed a variety of chemical components such as polysaccharides, saponins, and flavonoids, which possess a variety of biological activities such as antimicrobial, lipid-regulating, anti-aging, hypoglycemic, and anti-inflammatory. However, to date, the structure and activity of its oligosaccharide components are still unclear. In this study, we developed a method combining ultra-high-performance liquid chromatography with Q-Exactive Orbitrap mass spectrometry (UHPLC-Q-Exactive Orbitrap MS) and monosaccharide analysis for the identification of oligosaccharides in PCH. Finally, a total of 44 oligosaccharides, including 27 fructo-oligosaccharides (FOS), 10 arabino-oligosaccharides (AOS), and 7 others, were identified based on the precise relative molecular mass and fragment ion information provided by high-resolution mass spectrometry, in combination with standard comparison, monosaccharide composition analysis, and the relevant literature reports. All of those oligosaccharides were reported for the first time. These findings laid the foundation for the subsequent study of its medicinal substances and provided a theoretical basis for the more comprehensive development and utilization of PCH as a medicinal and edible product.

## 1. Introduction

*Polygonatum cyrtonema* Hua (PCH), a species belonging to the genus *Polygonatum* within the family *Liliaceae*, is widely distributed in the Hunan, Sichuan, Hubei, and Guizhou regions of China [1,2]. The rhizome of PCH is widely used as a folk medicine for anti-obesity [3], anti-fatigue [4], diabetes [5], Alzheimer’s disease [6], and cardiovascular diseases [7]. Previous studies have shown that PCH has a wide range of antimicrobial, lipid-regulating, anti-aging, hypoglycemic, and anti-inflammatory activities [8]. The potential health benefits of PCH may be attributed to its bioactive constituents, such as polysaccharides, saponins, and flavonoids, among other compounds. Interestingly, it has been shown that low-molecular-weight oligosaccharides hydrolyzed from polysaccharides also possess significant bioactivity, suggesting that oligosaccharides may be important functional components of PCH [9,10]. However, current studies on PCH mainly focus on polysaccharides and lack studies on its oligosaccharide components. Therefore, it is necessary to establish a systematic and rapid detection and identification method to further study the oligosaccharide components in PCH.

Oligosaccharides are defined as biomolecules consisting of two to ten monosaccharide units [11]. They are abundantly present in medicinal plants and play a crucial role, which is not only as energy storage components but also in the treatment of various diseases. Their biological activities are closely related to their monosaccharide composition, glycosidic bonding patterns, and branching properties [9]. In recent years, the relationship between the structure and activity of oligosaccharides has attracted the attention of an increasing number of researchers. In order to elucidate the relationship between the structure and activity of oligosaccharides, it is essential to analyze and characterize oligosaccharides first. The analysis of oligosaccharides has long relied on derivatization techniques to enhance their hydrophobicity, thereby improving performance in chromatographic separation and mass spectrometry detection [12,13]. However, the derivatization process is complex and time-consuming, with reaction efficiency influenced by various factors. This may lead to incomplete derivatization, affecting quantitative accuracy and detection sensitivity while introducing by-products or interfering substances that increase analytical complexity. In recent years, the application of ultra-performance liquid chromatography coupled with high-resolution tandem mass spectrometry (UPLC-HRMS) has significantly enhanced the accuracy and efficiency of oligosaccharide molecular weight determination, structural elucidation, and isomer differentiation without the need for derivatization steps [14,15]. Ultra-high-performance liquid chromatography-Q-Exactive Orbitrap mass spectrometry (UHPLC-Q-Exactive Orbitrap MS) enables comprehensive data acquisition through a full MS scan with data-dependent MS^2^ (full MS/ddMS^2^) mode. This mode simultaneously collects both MS1 (full MS) and MS2 (ddMS^2^) data, providing detailed molecular and structural information for complex sample analysis [16,17]. Among them, the parallel reaction monitoring (PRM) mode has the ability to extract trace ions from the mass spectra, particularly the parent ions that are eluted concurrently with components at high concentrations. So that trace amounts of the required compounds are not overlooked.

Ion chromatography (IC) efficiently separates monosaccharides by utilizing the anionic properties of carbohydrate compounds in high pH eluents, along with their pKa differences and hydrophobic interactions with anion exchange resins. The detection is achieved by measuring the current generated from the oxidation reaction of hydroxyl groups in sugar molecular structures occurring on the surface of a gold electrode [18,19]. This method provides a sensitive and efficient means for the analysis of monosaccharide composition in oligosaccharides. The analysis of monosaccharide composition clarifies the basic components of oligosaccharides, effectively distinguishing certain isomeric monosaccharides. It also provides crucial support for mass spectrometry data interpretation, serving as a key supplementary method that offers indispensable information for the accurate identification of oligosaccharide structures.

In this study, we first analyzed the monosaccharide composition of PCH using IC. Building upon this foundation, we systematically characterized 44 oligosaccharide components in PCH using UHPLC-Q-Exactive Orbitrap MS technology, with comparisons made against oligosaccharide standards and the literature data. All of those oligosaccharides were reported for the first time. These findings laid the foundation for the subsequent study of its medicinal substances and provided a theoretical basis for the more comprehensive development and utilization of PCH as a medicinal and edible product.

## 2. Results

### 2.1. Analytical Strategy

**Figure 1:**
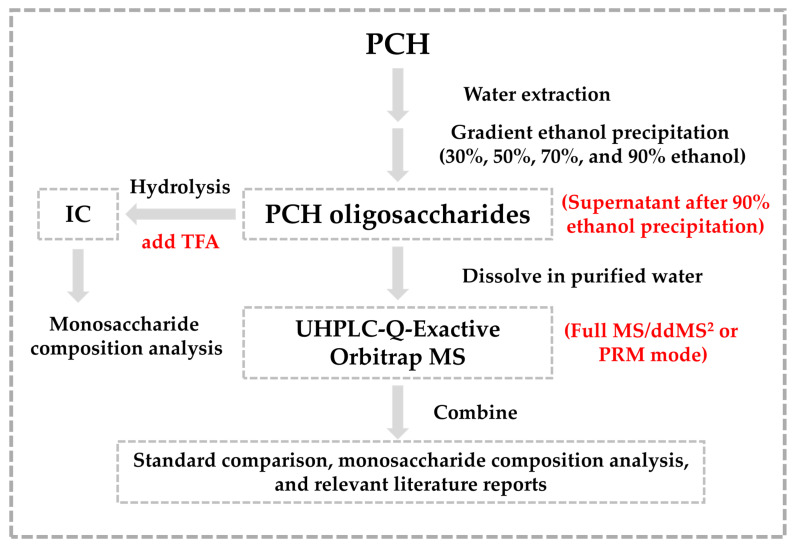
Novel strategy for PCH oligosaccharides characterization.

To comprehensively screen and identify oligosaccharides in PCH, this study established a combined strategy using monosaccharide composition analysis and UHPLC-Q-Exactive Orbitrap MS technology. Initially, PCH water extract was obtained through heating and reflux extraction. After concentration, a gradient ethanol precipitation was performed using 30%, 50%, 70%, and 90% ethanol to remove polysaccharides. The supernatant from the 90% ethanol precipitation was collected, concentrated, and freeze-dried to obtain PCH oligosaccharides. Secondly, the monosaccharide composition of PCH oligosaccharides was analyzed by IC after hydrolysis. Additionally, the non-hydrolyzed and non-derivatized oligosaccharides were directly analyzed using UHPLC-Q-Exactive Orbitrap MS in the full MS/ddMS² mode. For trace compounds that do not produce secondary fragment ions, MS^2^ data can be obtained by separating the target precursor ions and product fragment ions in PRM mode, allowing for a more adequate identification of oligosaccharides in PCH. Finally, the compounds were identified based on comparison with standards, summary diagnostic fragment ions (DFIs), neutral loss, and monosaccharide composition analysis, as well as through comparison with the literature. As shown in Figure 1, a novel strategy for the characterization of PCH oligosaccharides was developed.

### 2.2. Monosaccharide Composition

**Figure 2:**
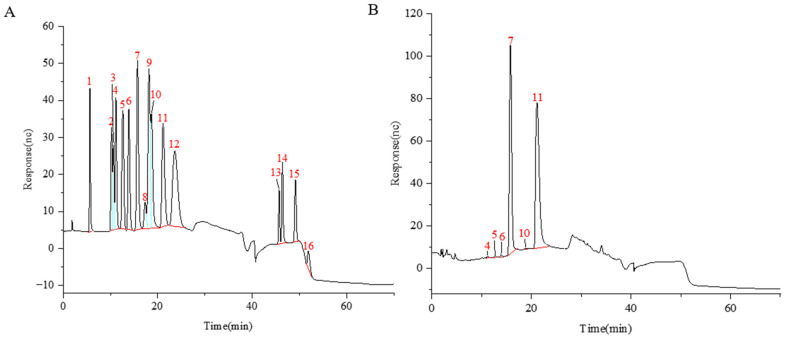
Ion chromatograms of monosaccharide standards (**A**) and PCH oligosaccharide sample (**B**). The peaks from 1 to 16 are as follows: fucose (Fuc), galactosamine hydrochloride (GalN), rhamnose (Rha), arabinose (Ara), glucosamine hydrochloride (GlcN), galactose (Gal), glucose (Glc), N-acetyl-D-glucosamine (GlcNAc), xylose (Xyl), mannose (Man), fructose (Fru), ribose (Rib), galacturonic acid (GalA), guluronic acid (GulA), glucuronic acid (GlcA), and mannuronic acid (ManA).

The PCH oligosaccharide sample obtained after gradient alcohol precipitation was hydrolyzed with trifluoroacetic acid (TFA) and subsequently analyzed using the Dionex ICS-5000 HPIC system (Thermo Fisher Scientific, Vacaville, CA, USA). The system is equipped with a Dionex CarboPac™ PA20 (Thermo Fisher Scientific, Vacaville, CA, USA, 3.0 mm × 150 mm) anion exchange column and utilizes an electrochemical detector for detection. The gradient elution method was used to separate and quantify 16 common monosaccharides (see Table S2 for details), successfully revealing the monosaccharide composition of PCH oligosaccharides, as shown in Figure 2. PCH oligosaccharides are composed of fructose (Fru), glucose (Glc), arabinose (Ara), galactose (Gal), glucosamine hydrochloride (GlcN), and mannose (Man).

### 2.3. Identification and Analysis of PCH Oligosaccharide Components

The LC-MS analyses were performed using a Dionex Ultimate 3000 RS UHPLC system equipped with a quaternary pump and an LPG-3400SD vacuum degasser unit (Thermo Fisher Scientific, Vacaville, CA, USA). The UHPLC system was coupled to a Q-Exactive Focus Orbitrap MS via an electrospray ionization (ESI) source (Thermo Electron, Bremen, Germany). The oligosaccharide sample obtained by the PCH gradient alcohol precipitation method was analyzed using UHPLC-Q-Exactive Orbitrap MS in negative ion mode. Firstly, the characteristic peaks observed in the total ion chromatogram acquired under the full MS/ddMS^2^ mode were initially identified. The peaks detected with an intensity of over 10,000 were selected for identification. By comparing and analyzing the MS/MS fragment ions with a self-constructed oligosaccharide database, we employed the PRM mode to perform targeted secondary mass spectrometry acquisition for compounds that exhibited insufficient fragment information. Based on the analysis of glycosidic bond cleavage and fragment ions, the sample was identified as containing pentose and hexose units. Finally, the structure of the oligosaccharides was determined by comparing their monosaccharide composition with PCH, oligosaccharide standards, and the literature data. Table 1 and Table S1 summarize the chromatographic and mass data of the assayed components, including retention times, experimental masses, discrepancies (in ppm) between theoretical and experimental masses, molecular formulas, and MS/MS fragment ion data. Figure S1 shows the high-resolution extracted ion chromatogram of the PCH oligosaccharide sample. All compounds were numbered according to their elution order.

#### 2.3.1. Identification of FOS

**Figure 3:**
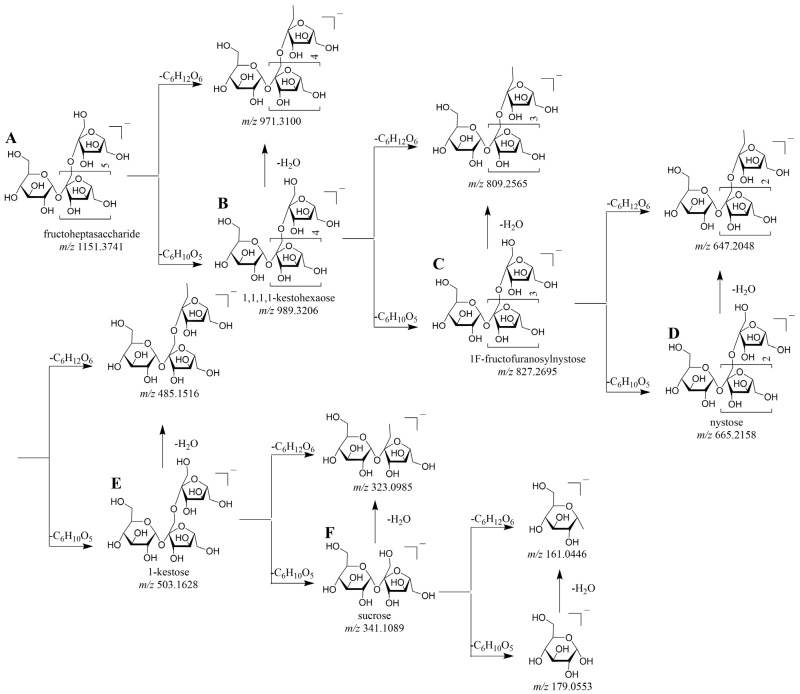
Fragmentation routes of the reference standards: fructoheptasaccharide (**A**); 1,1,1,1-kestohexaose (**B**); 1F-fructofuranosyl nystose (**C**); nystose (**D**); 1-kestose (**E**); sucrose (**F**).

The retention times of compounds **6**, **12**, **15**, and **17** were recorded as 18.38, 20.76, 21.16, and 21.73 min, respectively. The quasi-molecular ion peak [M − H]^−^ at *m/z* 503.1618 was detected for all these compounds. According to the fragmentation pathway illustrated in Figure 3E, the characteristic fragment ion at *m/z* 341.1089 was generated as a result of the loss of a Fru residue (162 Da) from the quasi-molecular ion. The ion at *m/z* 323.0985 was generated by the loss of one molecule of H_2_O (18 Da) from [M − C_6_H_10_O_5_]^−^. The *m/z* 179.0553 and 161.0446 correspond to the neutral losses of C_6_H_12_O_6_ (180 Da) and C_6_H_10_O_5_ (162 Da), respectively. Therefore, compound **15** was identified as 1-kestose, and the other compounds were isomers of 1-kestose [20]. Based on the comparative analysis of the diagnostic ions (*m/z* 485.1516, 341.1089, 323.0985, 179.0553, and 161.0446) shown in Figure 3D, along with retention time, compound **27** was identified as nystose. The compounds **22** and **30**, in comparison to nystose, demonstrate variations in retention time; however, they possess identical characteristic fragment ions. Based on the aforementioned analytical results and a previous literature report [21], the two compounds were identified as isomers of nystose. Compound **33** was identified as 1F-fructofuranosyl nystose by comparing its retention time and MS/MS fragmentation pattern with authentic reference standards. Compounds **5**, **7**, and **35** presented the same [M − H]^−^ ion at *m/z* 827.2674 (C_30_H_51_O_26_). Their MS^2^ spectra gave the expected MS^2^ ions at *m/z* 485.1521 (C_18_H_29_O_15_), 341.1088 (C_12_H_21_O_11_), 323.0976 (C_12_H_19_O_10_), 179.0551 (C_6_H_11_O_6_), and 161.0444 (C_6_H_9_O_5_), which attributed to the neutral loss of the hexose. As far as we know, such compounds have not previously been characterized definitively; therefore, they were considered as 1F-fructofuranosyl nystose isomers. Compound **36** was eluted at 24.94 min, resulting in the generation of a quasi-molecular ion peak [M − H]^−^ at *m/z* 989.3172. The secondary fragment ions *m/z* 827.2651, 665.2156, 503.1620, 341.1084, and 179.0550 are produced by the successive loss of a Fru residue from the quasi-molecular ion peak. Furthermore, fragment ions such as *m/z* 809.2600, 647.2039, 485.1523, 323.0978, and 161.0443 were observed; these ions are formed by the subsequent loss of one H_2_O molecule from their corresponding fragments. Based on the fragmentation patterns and diagnostic ions in Figure 3A, compounds **9**, **13**, and **39** were identified as fructoheptasaccharide and their isomers.

Based on the analysis of the series of cleavage pathways from sucrose to fructoheptasaccharide, we systematically summarize the DFIs generated by neutral losses. Therefore, for fructo-oligosaccharides (FOS) with a degree of polymerization greater than 7, preliminary identification primarily relies on the DFIs summarized previously and the characteristic MS/MS fragments reported in the literature [22]. Compounds **18** and **40** were preliminarily identified as fructo-oligosaccharide DP8/GF7; compounds **24** and **41** were identified as fructo-oligosaccharide DP9/GF8; compounds **28**, **37**, and **42** as fructo-oligosaccharide DP10/GF9; compounds **31**, **38**, and **43** as fructo-oligosaccharide DP11/GF10; compounds **34** and **44** as fructo-oligosaccharide DP12/GF11.

#### 2.3.2. Identification of AOS

Based on the monosaccharide composition, the only pentasaccharide in PCH oligosaccharides was composed of Ara. Compounds **2**, **4**, **11**, **14**, **20**, **26**, **32**, **16**, **19**, and **23** were known to be arabino-oligosaccharides (AOS) according to their characteristic fragment ions. Compounds **2**, **4**, **11**, and **14**, found at 14.08, 15.22, 20.51, and 21.11 min, showed a common precursor ion at [M − H]^−^ *m/z* 413.1301 (C_15_H_26_O_13_). They mainly yielded fragment ions at *m/z* 131.0337, 149.0441, and 281.1978 were tentatively characterized as arabinotriose or its isomers [23]. Compounds **20**, **26**, and **32** were eluted at 22.14, 22.62, and 23.63 min, respectively, having the same precursor ion [M − H]^−^ at *m/z* 545.1723, and the fragment ion at *m/z* 413.1938, attributed to the neutral loss of Ara residue (132 Da). These compounds were tentatively proposed as being arabinotetraose or its isomers [24]. Compounds **16**, **19**, and **23**, detected at 21.43, 21.95, and 22.33 min, respectively, possessed the same quasi-molecular ions [M − H]^−^ at *m/z* 809.2568, and the main daughter ion at *m/z* 527.1722, 395.1838, and 263.0953. They were deduced as being arabinohexaose or its isomers from the previous literature [24].

#### 2.3.3. Others

Compounds **1**, **3**, **8**, and **10** presented the same [M − H]^−^ ion at *m/z* 473.1512 (C_17_H_30_O_15_). They yielded *m/z* 179.0522 due to the loss of an Ara and Fru residue. As far as we know, such compounds have not previously been characterized unequivocally; therefore, they were considered 2Fru:1Ara. Compounds **21**, **25**, and **29** were found at 22.16, 22.60, and 23.17 min and showed a common precursor ion at [M − H]^−^ at *m/z* 575.1828 (C_21_H_36_O_18_). They yielded *m/z* 311.0977 due to the loss of two Ara residues (264 Da). They were tentatively identified as 3Ara:1Fru [25,26].

## 3. Discussion

Jian et al. employed HPLC-QTOF-MS/MS to identify and characterize saccharides in PCH during the steaming process, revealing the presence of substantial amounts of oligosaccharides composed of Fru units linked through β-(2→1) or β-(2→6) glycosidic bonds [20]. Lili et al. used FT-IR, MALDI-TOF-MS, NMR, AFM, and TEM to show that two oligosaccharides (PFOS-1 and PFOS-2) isolated from PCH are graminan-type fructan, with molar ratios of Glc to Fru being 1:5 and 1:9, respectively [9]. However, the specific composition and structural characteristics of PCH oligosaccharides remain insufficiently investigated, warranting further comprehensive studies to elucidate their detailed chemical profiles and potential bioactive properties. In this study, we obtained PCH oligosaccharide components using a water extraction and ethanol precipitation method. We developed a novel approach by combining UHPLC-Q-Exactive Orbitrap MS technology with IC for effective characterization of these oligosaccharides. Using the high sensitivity, resolution, and precise mass measurement capabilities of UHPLC-Q-Exactive Orbitrap MS, along with hydrophilic chromatography columns, we successfully achieved direct analysis of oligosaccharide samples, eliminating the need for complex derivatization processes. We use the XBridge^®^ BEH Amide column, which features a stable tridentate amide stationary phase that remains effective across a pH range of 2 to 11. This allows for efficient retention and separation of various polar compounds, including carbohydrates and sugars. Through LC-MS analysis, we have determined that the oligosaccharides are primarily composed of hexoses and pentoses. Based on the analysis of monosaccharide composition, we accurately identified the pentose sugar as Ara. Consequently, we identified that the pentose oligosaccharides in the LC-MS spectrum match the profile of AOS. For other oligosaccharide components, their structures were verified by comparing with standards in addition to inferring from mass spectrometry behavior. This multi-method strategy significantly enhances the accuracy and reliability of oligosaccharide structure identification. The characterized oligosaccharide components were further classified into 27 FOS, 10 AOS, and 7 other oligosaccharides.

FOS are oligosaccharides mainly composed of Fru units linked by β-(2→1) glycosidic bonds, typically starting with a Glc unit at the reducing end (Glc-Fru) and occasionally exhibiting minor β-(2→6) branching. Studies have demonstrated that FOS are widely recognized for their prebiotic properties, as they selectively stimulate the proliferation and activity of beneficial gut microbiota. FOS is recognized as a typical prebiotic that exerts beneficial effects on intestinal health. Chengcheng et al. found that FOS alleviates gut microbiota dysbiosis, regulates tryptophan metabolism in microbes, and promotes the production of indole-3-acetic acid (IAA) and indole-3-propionic acid (IPA). This activation triggers the aromatic hydrocarbon receptors (AhR)/interleukin-22 (IL-22) axis, effectively reducing colitis symptoms in mice induced by dextran sulfate sodium (DSS) [27]. Qin et al. [28] PCH oligosaccharide alleviated colitis symptoms by protecting the intestinal barrier, regulating short-chain fatty acids and inflammatory factors, and increasing *Faecalibaculum* abundance in mice. Oligosaccharides significantly attenuated inflammation in LPS-induced peritonitis mice [9] and alleviated tissue damage and inflammatory responses in NCM460 cells within a DSS-induced colitis model using C57BL/6J mice while simultaneously promoting the growth of probiotics [10].

AOS primarily consists of Ara, a five-carbon aldopentose, with its units typically linked by α-(1→5) glycosidic bonds in the main chain, while branching through α-(1→2) or α-(1→3) linkages introduces structural complexity [24]. AOS has the capacity to selectively promote the proliferation of beneficial gut microbiota, including species from the genera Bifidobacterium and Bacteroides, thereby contributing to various health-enhancing functions. AOS derived from sugar beet pulp has the potential to ameliorate inflammatory conditions in patients with ulcerative colitis (UC). This beneficial effect is mediated by the stimulation of bacteria that promote anti-inflammatory responses, as well as the production of acetate during the fermentation process [29]. The aforementioned research indicates that FOS and AOS, as typical prebiotics, exhibit significant biological activity. Based on the identified structural characteristics of these oligosaccharides, future studies could further explore their pharmacological effects, prebiotic properties, and interactions with the gut microbiota. This would contribute to a more comprehensive understanding of the specific molecular mechanisms by which oligosaccharides regulate gut microbial communities.

## 4. Materials and Methods

### 4.1. Materials and Reagents

The rhizome of PCH (20220607) was acquired from Hunan Bestcome Traditional Medicine Co., Ltd. (Huaihua, China). Detailed information on monosaccharides and oligosaccharide standards is provided in Table S2. TFA was obtained from Beijing InnoChem Science & Technology Co., Ltd. (Beijing, China). The reagents used for the mobile phase in this experiment were all purchased from Thermo Fisher Scientific Co., Ltd. (Fair Lawn, NJ, USA). These include a 50% sodium hydroxide solution, sodium acetate, MS-grade formic acid, LC-grade methanol, and LC grade acetonitrile. Purified water was sourced from Guangzhou Watsons Food & Beverage Co., Ltd. (Guangzhou, China). Other solvents were of an analytical grade.

### 4.2. PCH Oligosaccharide Extraction

First, the dried PCH samples were ground into a fine powder. Subsequently, they were mixed with pure water at a material ratio of 1:20 and refluxed at 80 °C for a duration of 2 h. Following three extraction cycles, the resulting extracts were combined and filtered. The extracts were then concentrated using a rotary evaporator under reduced pressure at 55 °C. The concentrated solution was subjected to precipitation using ethanol concentrations of 30%, 50%, 70%, and 90% in succession. The supernatant obtained after the final precipitation with 90% ethanol was collected. Subsequently, the supernatant was centrifuged at 3000 rpm for 20 min, after which it was further concentrated and lyophilized to yield PCH oligosaccharides.

### 4.3. Monosaccharide Composition Analysis

#### 4.3.1. Preparation of Monosaccharide Standards and PCH Solution

The 16 monosaccharide standards were accurately weighed and hydrolyzed using 2 mL of 3 mol/L TFA at 80 °C for 2 h. Nitrogen blowing drying, adding the purified water vortex mixing to obtain the stock standard solutions. Accurately measure the precisely configured concentrations of each monosaccharide standard solution and prepare a mixed standard sample. The PCH oligosaccharide (5 mg) was hydrolyzed and prepared according to the procedures outlined for the standard samples mentioned above. The aforementioned solution was subjected to centrifugation at 12,000 rpm for a duration of 5 min. The resulting supernatant was subsequently collected for IC analysis.

#### 4.3.2. Instruments and IC Conditions

Due to the electrochemical activity of sugar molecules and their ionization in strong alkaline solutions, the monosaccharide composition of PCH was analyzed using a Dionex ICS-5000 HPIC system chromatograph (Thermo Fisher Scientific Co., Ltd., Vacaville, CA, USA). A volume of 25 µL of the supernatant was utilized for analysis using an ion chromatograph equipped with an electrochemical detector and a Dionex Carbopac™ PA20 column (Thermo Fisher Scientific Co., Ltd., Vacaville, CA, USA). The flow rate of the chromatograph is set at 0.3 mL/min, and the column temperature is maintained at 30 °C. The mobile phases consisted of water (solvent A), 15 mM NaOH (solvent B), and 15 mM NaOH and 100 mM NaAc (solvent C). The gradient program was as follows: 0 min A/B/C (98.8:1.2:0, *v*/*v*), 18 min A/B/C (98.8:1.2:0, *v*/*v*), 20 min A/B/C (50:50:0, *v*/*v*), 30 min A/B/C (50:50:0, *v*/*v*), 30.1 min A/B/C (0:0:100, *v*/*v*), 46 min A/B/C (0:0:100, *v*/*v*), 46.1 min A/B/C (0:100:0, *v*/*v*), 50 min A/B/C (0:100:0, *v*/*v*), 50.1 min A/B/C (98.8:1.2:0, *v*/*v*), 80 min A/B/C (98.8:1.2:0, *v*/*v*).

### 4.4. LC-MS Analysis

#### 4.4.1. Preparation of Oligosaccharide Standards and PCH Solution

One milligram of an oligosaccharide (sucrose, 1-kestose, nystose, 1F-fructofuranosylnystose, 1,1,1,1-kestohexaose, and fructoheptasaccharide) was precisely weighed and dissolved in 1 mL of purified water. The obtained solution was centrifuged at 4 °C at 12,000 rpm for 10 min and then filtered through a syringe filter (0.22 μm of pore size). After diluting each monosaccharide standard to a concentration of 100 μg/mL, a specific volume of each was aliquoted to prepare a mixed standard. The PCH oligosaccharide (1 mg) was dissolved in purified water (1 mL) and centrifuged at 4 °C at 12,000 rpm for 10 min. A volume of 2 µL of supernatant was injected into the LC-MS system for further analysis.

#### 4.4.2. Instruments and LC-MS Conditions

The LC-MS analyses were performed using a Dionex Ultimate 3000 RS UHPLC, which is equipped with a quaternary pump and an LPG-3400SD vacuum degasser unit (Thermo Fisher Scientific, Vacaville, CA, USA). Additionally, we employ a Q-Exactive Focus Orbitrap Mass Spectrometer (Thermo Electron, Bremen, Germany) was utilized in conjunction with an ESI source. Chromatographic separation for all samples was conducted utilizing an XBridge^®^ BEH Amide column (Waters, Milford, MA, USA, 250 × 4.6 mm, 5 µm), which was maintained at a temperature of 40 °C. The mobile phase comprises a 0.1% aqueous solution of formic acid (solvent A) and acetonitrile (solvent B). It is delivered at a constant flow rate of 0.4 mL/min to achieve the best separation effect. The gradient program was as follows: 0 min, 80% B; 15 min, 57% B; 18 min, 50% B; 35 min, 50% B; 35.1 min, 80% B; 40 min, 80% B. The total runtime was 40 min with a sample injection volume of 2 µL.

MS analysis was conducted in negative ionization mode utilizing ESI, covering a scan range of *m/z* 150–2000. The parameters for the ESI source were configured as follows: The spray voltage was set to 3.2 kV; sheath gas and auxiliary gas flow rates were established at 35 and 10 (arbitrary units), respectively; the capillary temperature was maintained at 320 °C; the auxiliary gas heater temperature was adjusted to 350 °C; and the RF level of the S-lens was calibrated to 60. The full MS scan mode generates high-resolution mass spectra, achieving a resolution of 70,000. The MS^2^ data, acquired at a resolution of 17,500, was obtained through ddMS^2^ scanning or PRM mode triggered by an inclusion ions list generated from molecular predictions. The isolation window was set to 3.0 *m*/*z*, with a normalized collision energy (NCE) of 30% and an automatic gain control (AGC) target value of 5.0 × 10^4^, using nitrogen (purity ≥ 99.999%) as the collision gas to generate fragment ions. 

## 5. Conclusions

In this study, we systematically characterized the oligosaccharides in PCH using UHPLC-Q-Exactive Orbitrap MS. A total of 44 oligosaccharides were successfully identified through monosaccharide composition analysis, standard comparison, and mass spectrometry fragmentation pattern analysis. This includes 27 FOS, 10 AOS, and 7 others. All of those oligosaccharides were reported for the first time. By analyzing oligosaccharide structures in detail, we clarified their cleavage patterns and characteristic fragment profiles, providing a theoretical basis and data support for subsequent high-throughput identification, structural analysis, and bioactivity research. These findings established a foundation for further research on its medicinal components and offered a theoretical basis for the comprehensive development of PCH as both a medicinal and edible resource.

## Figures and Tables

**Table 1:** The chromatographic and mass data for the components detected from PCH though UHPLC-Q-Exactive Orbitrap MS.

Peak	t_R_ (min)	Theoretical Mass *m*/*z*	Experimental Mass *m*/*z*	Error (ppm)	Formula [M − H]^−^	Identification
1 ****	14.06	473.1512	473.1493	−3.96	C_17_H_30_O_15_	2Fru:1Ara
2 ***	14.08	413.1301	413.1289	−2.87	C_15_H_26_O_13_	arabinotriose
3 ****	14.81	473.1512	473.1517	1.13	C_17_H_30_O_15_	2Fru:1Ara
4 ***	15.22	413.1301	413.1291	−2.29	C_15_H_26_O_13_	arabinotriose
5 ***	18.03	827.2674	827.2657	−2.07	C_30_H_52_O_26_	1F-fructofuranosyl nystose isomer
6 ***	18.38	503.1618	503.1604	−2.64	C_18_H_32_O_16_	1-kestose isomer
7 ***	18.41	827.2674	827.2655	−2.30	C_30_H_52_O_26_	1F-fructofuranosyl nystose isomer
8 ****	20.11	473.1512	473.1489	−4.78	C_17_H_30_O_15_	2Fru:1Ara
9 ***	20.35	1151.3730	1151.3700	−2.65	C_42_H_72_O_36_	fructoheptasaccharide isomer
10 ****	20.46	473.1512	473.1493	−4.02	C_17_H_30_O_15_	2Fru:1Ara
11 ***	20.51	413.1301	413.1286	−3.62	C_15_H_26_O_13_	arabinotriose
12 ***	20.76	503.1618	503.1603	−2.88	C_18_H_32_O_16_	1-kestose isomer
13 ***	20.89	1151.3730	1151.3701	−2.55	C_42_H_72_O_36_	fructoheptasaccharide isomer
14 ***	21.11	413.1301	413.1289	−2.87	C_15_H_26_O_13_	arabinotriose
15 *	21.16	503.1618	503.1603	−2.88	C_18_H_32_O_16_	1-kestose
16 ***	21.43	809.2568	809.2546	−2.79	C_30_H_50_O_25_	arabinohexaose
17 ***	21.73	503.1618	503.1602	−3.00	C_18_H_32_O_16_	1-kestose isomer
18 ***	21.76	1313.4258	1313.4222	−2.77	C_48_H_82_O_41_	fructo-oligosaccharide DP8/GF7
19 ***	21.95	809.2568	809.2551	−2.11	C_30_H_50_O_25_	arabinohexaose
20 ***	22.14	545.1723	545.1708	−2.83	C_20_H_34_O_17_	arabinotetraose
21 ****	22.16	575.1828	575.1813	−2.81	C_21_H_36_O_18_	3Ara:1Fru
22 ***	22.16	665.2146	665.2130	−2.36	C_24_H_42_O_21_	nystose isomer
23 ***	22.33	809.2568	809.2545	−2.94	C_30_H_50_O_25_	arabinohexaose
24 ***	22.33	1475.4787	1475.4745	−2.85	C_54_H_92_O_46_	fructo-oligosaccharide DP9/GF8
25 ****	22.60	575.1828	575.1813	−2.69	C_21_H_36_O_18_	3Ara:1Fru
26 ***	22.62	545.1723	545.1707	−3.07	C_20_H_34_O_17_	arabinotetraose
27 *	22.73	665.2146	665.2129	−2.54	C_24_H_42_O_21_	nystose
28 ***	23.14	1637.5315	1637.5239	−4.64	C_60_H_102_O_51_	fructo-oligosaccharide DP10/GF9
29 ****	23.17	575.1828	575.1841	2.07	C_21_H_36_O_18_	3Ara:1Fru
30 ***	23.17	665.2146	665.2127	−2.81	C_24_H_42_O_21_	nystose isomer
31 ***	23.41	1799.5843	1799.5798	−2.51	C_66_H_112_O_56_	fructo-oligosaccharide DP11/GF10
32 ***	23.63	545.1723	545.1078	−2.83	C_20_H_34_O_17_	arabinotetraose
33 *	24.15	827.2674	827.2652	−2.67	C_30_H_52_O_26_	1F-fructofuranosyl nystose
34 ***	24.28	1961.6371	1961.6267	−5.33	C_72_H_122_O_61_	fructo-oligosaccharide DP12/GF11
35 ***	24.64	827.2674	827.2650	−2.90	C_30_H_52_O_26_	1F-fructofuranosyl nystose isomer
36 *	24.94	989.3202	989.3172	−3.06	C_36_H_62_O_31_	1,1,1,1-kestohexaose
37 ***	24.97	1637.5315	1637.5234	−4.94	C_60_H_102_O_51_	fructo-oligosaccharide DP10/GF9
38 ***	25.41	1799.5843	1799.5691	−8.48	C_66_H_112_O_56_	fructo-oligosaccharide DP11/GF10
39 *	25.66	1151.3730	1151.3694	−3.19	C_42_H_72_O_36_	fructoheptasaccharide
40 ***	26.46	1313.4258	1313.4219	−3.04	C_48_H_82_O_41_	fructo-oligosaccharide DP8/GF7
41 ***	27.04	1475.4787	1475.4747	−2.69	C_54_H_92_O_46_	fructo-oligosaccharide DP9/GF8
42 ***	27.65	1637.5315	1637.5267	−2.93	C_60_H_102_O_51_	fructo-oligosaccharide DP10/GF9
43 ***	28.32	1799.5843	1799.5786	−3.19	C_66_H_112_O_56_	fructo-oligosaccharide DP11/GF10
44 ***	28.92	1961.6371	1961.6313	−2.97	C_72_H_122_O_61_	fructo-oligosaccharide DP12/GF11

Refer to MSI standards, * denotes Level 1, *** denotes Level 3, **** denotes Level 4.

## Data Availability

The original contributions presented in this study are included in the article/Supplementary Materials. Further inquiries can be directed to the corresponding authors.

## Supplementary Materials

The following supporting information can be downloaded at https://www.mdpi.com/article/10.3390/molecules30071433/s1, Table S1: The chromatographic and mass data for the components detected from PCH through UHPLC-Q-Exactive Orbitrap MS. Table S2: Detailed information on monosaccharides and fructo-oligosaccharide standards. Figure S1: The high-resolution extracted ion chromatogram (HREIC) in 10 ppm for the multiple compounds in PCH. Peak 1, 3, 8, 10: *m/z* 473.1512; 2, 4, 11, 14: *m/z* 413.1301; 20, 26, 32: *m/z* 545.1723; 21, 25, 29: *m/z* 575.1828; 31, 38, 43: *m/z* 1799.5843; 34, 44: *m/z* 1961.6371; 36: *m/z* 989.3202 (A); Peak 16, 19, 23: *m/z* 809.2568; 24: *m/z* 1475.4787; 28, 37, 42: *m/z* 1637.5315; 41: *m/z* 1475.4787 (B); Peak 5, 7, 33, 35: *m/z* 827.2674; 6, 12, 15, 17: *m/z* 503.1618; 9, 13, 39: *m/z* 1151.3730; 18, 40: *m/z* 1313.4258; 22, 27, 30: *m/z* 665.2146 (C). Figure S2: MS^2^ spectra of the reference standards: Fructoheptasaccharide (A); 1,1,1,1-kestohexaose (B); 1F-fructofuranosyl nystose (C); Nystose (D); 1-kestose (E); Sucrose (F).
