# Supplementary material for: A Systematic Method for the Identification of Oligosaccharide Constituents in Polygonatum cyrtonema Hua Using UHPLC-Q-Exactive Orbitrap Mass Spectrometry
Source: Molecules. 2025 Mar 24;30(7):1433. doi: 10.3390/molecules30071433 (PMC11990173; doi:10.3390/molecules30071433)
Supplement: Supplementary file 1 [file molecules-30-01433-s001.zip › molecules-3504673-supplementary.pdf]

Table S1. The chromatographic and mass data for the components detected from PCH though UHPLC-Q-Exactive Orbitrap MS.

Peak	t_R(min)	Theoretical Mass m/z	Experimental Mass m/z	Error (ppm)	Formula [M-H]$^-$	MS/MS fragment	Identification
1****	14.06	473.1512	473.1493	-3.96	$C_{17}H_{30}O_{15}$	MS^2[473]:149.0445(100),89.0229(30),161.0442(10),131.0335(9),179.0552(4)	2Fru:1Ara
2***	14.08	413.1301	413.1289	-2.87	$C_{15}H_{26}O_{13}$	MS^2[413]:89.0231(100),149.0441(25),121.0281(20),293.0032(5),131.0337(2),113.0229(2),281.1978(20)	Arabinotriose
3****	14.81	473.1512	473.1517	1.13	$C_{17}H_{30}O_{15}$	MS^2[473]:89.0229(100),131.0338(58),161.0442(10),179.0552(5),149.0445(5)	2Fru:1Ara
4***	15.22	413.1301	413.1291	-2.29	$C_{15}H_{26}O_{13}$	MS^2[413]:89.0232(100),149.0445(25),281.1978(20),121.0281(20),311.2082(5),293.0032(5),131.0337(2), 113.0230(2)	Arabinotriose
5***	18.03	827.2674	827.2657	-2.07	$C_{30}H_{52}O_{26}$	MS^2[827]:161.0444(16),179.0551(100),323.0976(6),485.1521(5),113.0233(5),149.0442(5),707.2241(3),131.0337(3),341.1088(2)	1F-fructofuranosyl nystose isomer
6***	18.38	503.1618	503.1604	-2.64	$C_{18}H_{32}O_{16}$	MS^2[503]:89.0228(100),113.0230(26),161.0442(16),323.0986(6),149.0444(3),179.0550(4),221.0658(4),131.0336(3),341.1086(2)	1-kestose isomer
7***	18.41	827.2674	827.2655	-2.30	$C_{30}H_{52}O_{26}$	MS^2[827]:179.0551(100),161.0445(11),323.0976(6),113.0233(6),149.0442(5),341.1086(2),707.2240(3),131.0337(3),485.1521(2)	1F-fructofuranosyl Nystose isomer
8****	20.11	473.1512	473.1489	-4.78	$C_{17}H_{30}O_{15}$	MS^2[473]:89.0229(100),131.0336(26),161.0442(10),179.0552(5),149.0445(5)	2Fru:1Ara

Table S1. Cont.

Peak	t_R(min)	Theoretical Mass m/z	Experimental Mass m/z	Error (ppm)	Formula [M-H]$^-$	MS/MS fragment	Identification
9***	20.35	1151.3730	1151.3700	-2.65	$C_{42}H_{72}O_{36}$	MS^2[1151]:1151.3720(100),341.1084(90),179.0550(85),503.1620(38),1031.3326(10),323.0977(17),161.0440(15),485.1523(10),666.2175(7),665.2152(7),131.0337(5),827.2637(5),989.3228(5),647.2039(2),809.2600(2),113.0232(2),149.0445(2)	fructoheptasaccharide isomer
10****	20.46	473.1512	473.1493	-4.02	$C_{17}H_{30}O_{15}$	MS^2[473]:89.0229(100),131.0339(20),161.0442(10),149.0445(5),179.0552(4)	2Fru:1Ara
11***	20.51	413.1301	413.1286	-3.62	$C_{15}H_{26}O_{13}$	MS^2[413]:89.0230(100),149.0445(25),281.1977(20),121.0281(20),293.0032(10),131.0335(2),113.0230(2)	Arabinotriose
12***	20.76	503.1618	503.1603	-2.88	$C_{18}H_{32}O_{16}$	MS^2[503]:89.0228(100),113.0230(26),161.0442(16),323.0986(6),149.0444(3),179.0550(4),221.0658(4),131.0336(3),341.1086(2)	1-kestose isomer
13***	20.89	1151.3730	1151.3701	-2.55	$C_{42}H_{72}O_{36}$	MS^2[1151]:1151.3720(100),179.0550(85),341.1084(90),503.1620(38),323.0975(20),161.0445(11),1031.3326(10),485.1523(10),989.3228(8),666.2176(7),665.2155(7),827.2637(5),809.2600(2),647.2039(2),113.0232(2),149.0445(2)	fructoheptasaccharide isomer
14***	21.11	413.1301	413.1289	-2.87	$C_{15}H_{26}O_{13}$	MS^2[413]:89.0230(100),149.0442(25),281.1977(20),121.0281(20),293.0032(5),131.0337(2),113.0230(2)	Arabinotriose
15*	21.16	503.1618	503.1603	-2.88	$C_{18}H_{32}O_{16}$	MS^2[503]:89.0230(100),113.0230(26),179.0550(10),149.0444(3),323.0986(6),341.1086(6),221.0658(4),161.0446(4),131.0336(3)	1-kestose

Table S1. Cont.

Peak	t_R(min)	Theoretical Mass m/z	Experimental Mass m/z	Error (ppm)	Formula [M-H]$^-$	MS/MS fragment	Identification
16***	21.43	809.2568	809.2546	-2.79	$C_{30}H_{50}O_{25}$	MS^2[809]:89.0229(100),263.0953(40),131.0335(22),121.0289(20),149.0443(18),293.0032(5),395.1838(2),413.1938(2),527.1722(2)	Arabinohexaose
17***	21.73	503.1618	503.1602	-3.00	$C_{18}H_{32}O_{16}$	MS^2[503]:89.0229(100),113.0230(26),161.0443(6),179.0550(10),323.0986(6),149.0444(3),131.0336(3),221.0658(2),341.1086(2)	1-kestose isomer
18***	21.76	1313.4258	1313.4222	-2.77	$C_{48}H_{82}O_{41}$	MS^2[1313]:1313.4242(100),341.1086(6),179.0551(4),503.1620(4),665.2142(4),827.2637(4),989.3215(3),1151.3699(2),323.0981(2),485.1523(2),161.0445(2),113.0230(2)	Fructo-oligosaccharide DP8/GF7
19***	21.95	809.2568	809.2551	-2.11	$C_{30}H_{50}O_{25}$	MS^2[809]:89.0229(100),131.0335(22),263.0955(20),121.0289(20),149.0443(18),293.0034(5),395.1838(2),413.1938(2),527.1722(2)	Arabinohexaose
20***	22.14	545.1723	545.1708	-2.83	$C_{20}H_{34}O_{17}$	MS^2[545]:89.0230(100),263.0953(40),131.0337(22),121.0281(20),149.0442(18),293.0032(5),395.1838(2), 413.1938(2)	Arabinotetraose
21****	22.16	575.1828	575.1813	-2.81	$C_{21}H_{36}O_{18}$	MS^2[575]:89.0229(100),179.0550(45),121.0281(20),161.0443(13),311.0977(5),293.0032(5),131.0336(5), 149.0442(4)	3Ara:1Fru
22***	22.16	665.2146	665.2130	-2.36	$C_{24}H_{42}O_{21}$	MS^2[665]:179.0550(100),89.0229(50),161.0444(18),113.0230(13),323.0986(6),485.1478(5),149.0441(5),383.1196(3),131.0338(3),341.1086(2),545.1726(2)	Nystose isomer

Table S1. Cont.

Peak	t_R(min)	Theoretical Mass m/z	Experimental Mass m/z	Error (ppm)	Formula [M-H]-	MS/MS fragment	Identification
23***	22.33	809.2568	809.2545	-2.94	$C_{30}H_{50}O_{25}$	MS²[809]:89.0229(100),263.0953(40),131.0335(22),293.0032(19),149.0443(18),121.0289(10),395.1838(2),413.1938(2),527.1722(2)	Arabinohexaose
24***	22.33	1475.4787	1475.4745	-2.85	$C_{54}H_{92}O_{46}$	MS²[1475]:1313.4233(100),827.2637(55),665.2142(49),989.3224(48),1151.3717(44),341.1084(37),503.1614(27), 179.0549(23),485.1524(7),161.0443(2),323.0978(2),647.2039(2),809.2600(2),1133.3638(2),1295.4154(2)	Fructo-oligosaccharide DP9/GF8
25****	22.60	575.1828	575.1813	-2.69	$C_{21}H_{36}O_{18}$	MS²[575]:89.0229(100),179.0552(45),121.0281(20),161.0443(13),311.0977(5),131.0336(5),149.0442(5), 293.0032(5)	3Ara:1Fru
26***	22.62	545.1723	545.1707	-3.07	$C_{20}H_{34}O_{17}$	MS²[545]:89.0230(100),263.0953(40),131.0337(22),121.0281(20),149.0442(18),293.0032(5),395.1838(2), 413.1938(2)	Arabinotetraose
27*	22.73	665.2146	665.2129	-2.54	$C_{24}H_{42}O_{21}$	MS²[665]:179.0550(100),89.0229(78),113.0230(13),323.0980(6),485.1478(5),161.0443(5),149.0441(5),383.1196(3),131.0338(3),341.1081(2),545.1724(2)	Nystose
28***	23.14	1637.5315	1637.5239	-4.64	$C_{60}H_{102}O_{51}$	MS²[1637]:1637.5281(100),1475.4773(60),1151.3714(29),989.3220(26),827.2634(17),1313.4234(14),665.2148(11), 341.1084(7),161.0443(5),179.0549(5),503.1620(5),323.0978(2),485.1524(2),647.2039(2),809.2600(2),1133.3639(2),1295.4154(2),1457.4459(2)	Fructo-oligosaccharide DP10/GF9

Table S1. Cont.

Peak	t_R(min)	Theoretical Mass m/z	Experimental Mass m/z	Error (ppm)	Formula [M-H]$^-$	MS/MS fragment	Identification
29****	23.17	575.1828	575.1841	2.07	$C_{21}H_{36}O_{18}$	MS²[575]:89.0229(100),179.0550(45),161.0443(13),121.0281(10),311.0976(5),131.0336(5),293.0032(5), 149.0442(4)	3Ara:1Fru
30***	23.17	665.2146	665.2127	-2.81	$C_{24}H_{42}O_{21}$	MS²[665]:89.0229(100),179.0550(77),161.0444(28),113.0230(15),323.0986(6),485.1475(5),383.1196(5),149.0442(5),131.0338(3),341.1086(2),545.1726(2)	Nystose isomer
31***	23.41	1799.5843	1799.5798	-2.51	$C_{66}H_{112}O_{56}$	MS²[1799]:1799.5809(100),1151.3717(78),1475.4773(60),1637.5286(20),665.2144(20),1313.4234(14),1295.4155(10),827.2636(10),341.1084(7),989.3225(5),503.1620(5),179.0547(3),161.0443(2),323.0978(2),485.1524(2),647.2039(2),809.2600(2),1133.3639(2),1457.4459(2)	Fructo-oligosaccharide DP11/GF10
32***	23.63	545.1723	545.1078	-2.83	$C_{20}H_{34}O_{17}$	MS²[545]:89.0229(100),263.0953(20),149.0442(10),131.0337(7),395.1838(5),121.0280(5),413.1938(2),293.0032(2)	Arabinotetraose
33*	24.15	827.2674	827.2652	-2.67	$C_{30}H_{52}O_{26}$	MS²[827]:179.0551(100),341.1082(19),161.0445(18),323.0976(5),113.0233(5),149.0442(5),131.0337(3), 707.2241(3),485.1521(2),	1F-fructofuranosyl nystose
34***	24.28	1961.6371	1961.6267	-5.33	$C_{72}H_{122}O_{61}$	MS²[1961]:1799.5798(100),1637.5282(15),1295.4155(10),1475.4776(6),989.3225(5),1313.4237(5),1781.5852(4), 1133.3639(2),1151.3718(2)	Fructo-oligosaccharide DP12/GF11

Table S1. Cont.

Peak	t_R(min)	Theoretical Mass m/z	Experimental Mass m/z	Error (ppm)	Formula [M-H]$^-$	MS/MS fragment	Identification
35***	24.64	827.2674	827.2650	-2.90	$C_{30}H_{52}O_{26}$	MS^2[827]:179.0550(100),161.0444(15),341.1083(10),323.0976(6),113.0230(6),149.0442(5),131.0338(3),707.2241(3),485.1521(2),	1F-fructofuranosyl nystose isomer
36*	24.94	989.3202	989.3172	-3.06	$C_{36}H_{62}O_{31}$	MS^2[989]:179.0550(100),341.1084(38),161.0443(11),665.2156(7),323.0978(6),503.1620(5),131.0337(5),707.2234(5),485.1523(3),647.2039(2),809.2600(2),827.2651(2),869.2780(2),113.0230(2),149.0445(2)	1,1,1,1-kestohexaose
37***	24.97	1637.5315	1637.5234	-4.94	$C_{60}H_{102}O_{51}$	MS^2[1637]:1637.5281(100),1475.4772(60),1151.3714(29),989.3220(26),827.2634(17),1313.4231(14),665.2140(11),161.0443(12),179.0547(12),323.0978(7),341.1084(7),503.1628(5),485.1524(3),647.2039(2),809.2600(2),1133.3639(2),1295.4154(2),1457.4459(2)	Fructo-oligosaccharide DP10/GF9
38***	25.41	1799.5843	1799.5691	-8.48	$C_{66}H_{112}O_{56}$	MS^2[1799]:1799.5809(100),1151.3717(78),1475.4773(60),503.1620(35),1637.5286(25),665.2144(20),809.2602(20),179.0547(19),1313.4234(14),1295.4155(10),827.2636(10),341.1084(7),1133.3639(6),161.0443(5),323.0979(3),485.1524(3),647.2039(2),989.3220(5),1457.4459(2)	Fructo-oligosaccharide DP11/GF10
39*	25.66	1151.3730	1151.3694	-3.19	$C_{42}H_{72}O_{36}$	MS^2[1151]:1151.3720(100),341.1084(90),179.0550(85),503.1620(38),323.0977(17),161.0442(11),1031.3321(10),485.1523(10),666.2176(7),665.2150(7),827.2637(5),809.2600(2),989.3228(2),647.2039(2),113.0232(2),149.0442(2)	Fructoheptasaccharide

Table S1. Cont.

Peak	t_R(min)	Theoretical Mass m/z	Experimental Mass m/z	Error (ppm)	Formula [M-H]-	MS/MS fragment	Identification
40***	26.46	1313.4258	1313.4219	-3.04	$C_{48}H_{82}O_{41}$	MS²[1313]:665.2138(100),179.0550(26),341.1081(31),503.1614(4),827.2637(4),989.3215(3),1151.3699(2),161.0445(2),323.0981(2),485.1523(2),113.0232(2)	Fructo-oligosaccharide DP8/GF7
41***	27.04	1475.4787	1475.4747	-2.69	$C_{54}H_{92}O_{46}$	MS²[1475]:1313.4233(100),151.3717(44),485.1524(7),341.1084(3),161.0443(2),179.0553(2),323.0978(2),503.1614(2),647.2039(2),665.2144(4),809.2600(2),827.2637(5),989.3224(2),1133.3638(2),11295.4154(2)	Fructo-oligosaccharide DP9/GF8
42***	27.65	1637.5315	1637.5267	-2.93	$C_{60}H_{102}O_{51}$	MS²[1637]:1637.5281(100),1475.4773(60),1151.3714(29),989.3220(26),665.2144(20),1313.4234(14),341.1084(7),503.1620(5),179.0549(3),161.0443(2),323.0978(2),485.1524(2),647.2039(2),809.2600(2),827.2638(2),1133.3639(2),1295.4154(2),1457.4459(2)	Fucto-oligosaccharide DP10/GF9
43***	28.32	1799.5843	1799.5786	-3.19	$C_{66}H_{112}O_{56}$	MS²[1799]:1799.5804(100),1151.3717(78),827.2638(66),1475.4773(60),665.2140(48),341.1084(25),1637.5286(20),179.0547(16),503.1619(15),1295.4155(10),1313.4234(14),989.3225(5),323.0978(2),485.1524(2),647.2039(2),809.2600(2),1133.3639(2),1457.4459(2),161.0443(2)	Fructo-oligosaccharide DP11/GF10
44***	28.92	1961.6371	1961.6313	-2.97	$C_{72}H_{122}O_{61}$	MS²[1961]:1799.5803(100),1637.5285(10),1295.4155(10),1475.4779(7),989.3225(5),1313.4230(4),1781.5852(4),1133.3639(2),1151.3718(2)	Fructo-oligosaccharide DP12/GF11

Refer to MSI standards, * denotes Level 1, *** denotes Level 3,**** denotes Level 4.

Table S2: Detailed information on monosaccharides and fructo-oligosaccharide standards.

Name	Formula	Purity	Lot Number	Company
Mannose (Man)	$C_6H_{12}O_6$	98%	C17D9H77586	Bo Rui Saccharide Biotech Co. Ltd.
Rhamnose (Rha)	$C_6H_{12}O_5$	98%	H10S9Z69863	Bo Rui Saccharide Biotech Co. Ltd.
Galacturonic acid (GalA)	$C_6H_{10}O_7$	97%	K02A9B66077	Bo Rui Saccharide Biotech Co. Ltd.
Galactose (Gal)	$C_6H_{12}O_6$	99%	E1927035	Bo Rui Saccharide Biotech Co. Ltd.
Glucose (Glc)	$C_6H_{12}O_6$	99%	Q18F10N80946	Bo Rui Saccharide Biotech Co. Ltd.
Glucuronic acid (GlcA)	$C_6H_{10}O_7$	≥98%	K14M10S82777	Bo Rui Saccharide Biotech Co. Ltd.
Arabinose (Ara)	$C_5H_{10}O_5$	98%	S15A10G85850	Bo Rui Saccharide Biotech Co. Ltd.
Xylose (Xyl)	$C_5H_{10}O_5$	99%	A22S6X3606	Bo Rui Saccharide Biotech Co. Ltd.
Fucose (Fuc)	$C_6H_{12}O_5$	98%	X29D7Y27768	Bo Rui Saccharide Biotech Co. Ltd.
Glucosamine hydrochloride (GlcN)	$C_6H_{14}ClNO_5$	98%	A22S6X3606	Bo Rui Saccharide Biotech Co. Ltd.
N-acetyl-D-glucosamine (GlcNAc)	$C_8H_{15}NO_6$	98%	A21J8X40372	Bo Rui Saccharide Biotech Co. Ltd.
D-fructose (Fru)	$C_6H_{12}O_6$	99%	J01J10R89818	Bo Rui Saccharide Biotech Co. Ltd.
D-Ribose (Rib)	$C_5H_{10}O_5$	99%	H26F10Z81556	Bo Rui Saccharide Biotech Co. Ltd.
Galactosamine hydrochloride (GalN)	$C_6H_{14}ClNO_5$	98%	B01J8S37079	Bo Rui Saccharide Biotech Co. Ltd.
L-guluronic acid (GulA)	$C_6H_{10}O_7$	≥98%	S200115AG1	Bo Rui Saccharide Biotech Co. Ltd.
D-mannuronic acid (ManA)	$C_6H_{10}O_7$	≥98%	S200108AM1	Bo Rui Saccharide Biotech Co. Ltd.
Sucrose	$C_{12}H_{22}O_{11}$	≥98%	MUST-23082505	Chengdu Must Bio-Technology Co., Ltd.
1-Kestose	$C_{18}H_{32}O_{16}$	≥98%	MUST-23121113	Chengdu Must Bio-Technology Co., Ltd.
Nystose	$C_{24}H_{42}O_{21}$	92.2%	111891-201704	National Institutes for Food and Drug Control
1F-Fructofuranosyl nystose	$C_{30}H_{52}O_{26}$	94.2%	111965-201501	National Institutes for Food and Drug Control
1,1,1,1-Kestohexaose	$C_{36}H_{62}O_{31}$	≥98%	MUST-23110315	Chengdu Must Bio-Technology Co., Ltd.
Fructoheptasaccharide	$C_{42}H_{72}O_{36}$	≥98%	MUST-24070501	Chengdu Must Bio-Technology Co., Ltd.

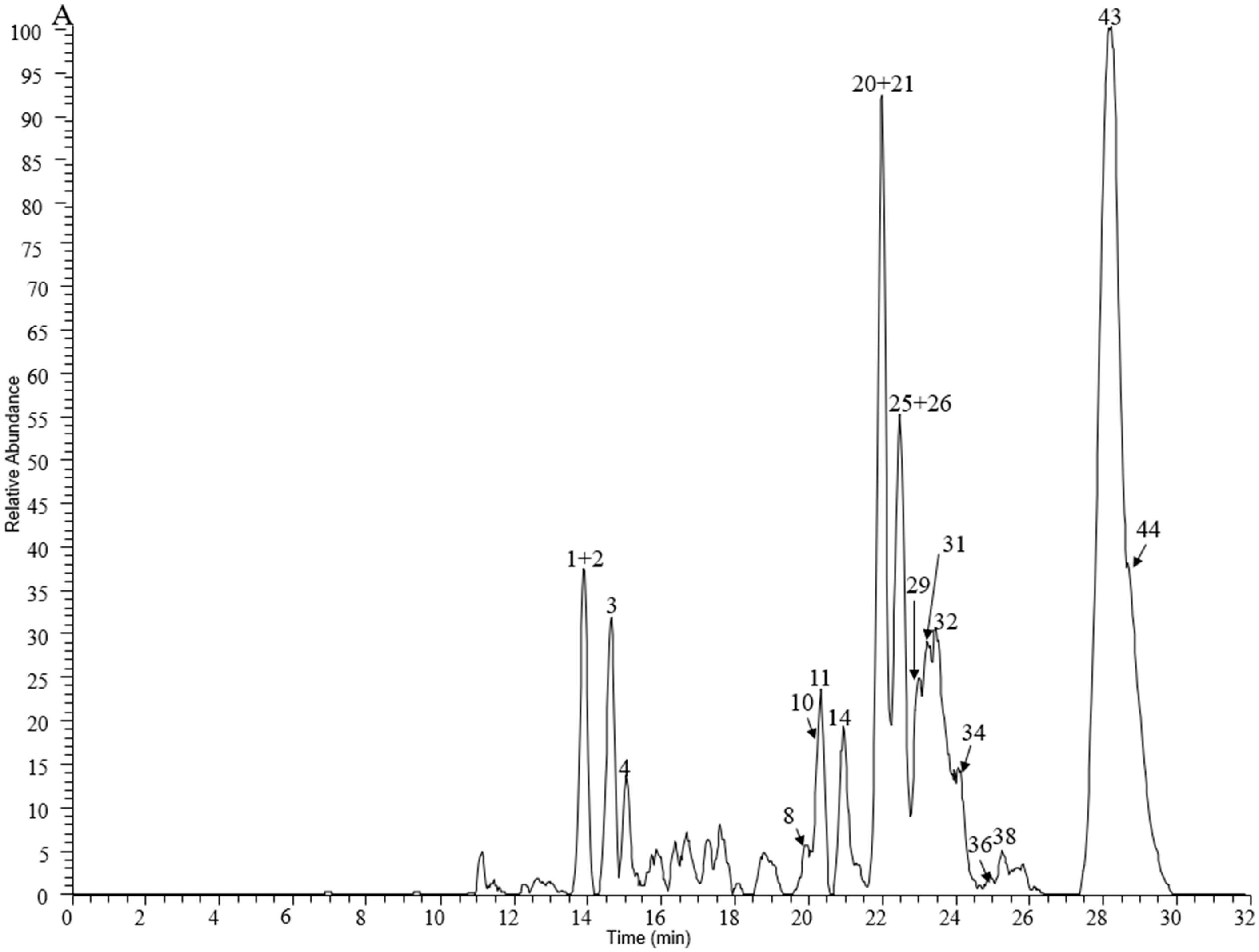
A
Relative Abundance
Time (min)
1+2
3
4
8
10
11
14
20+21
25+26
29
31
32
34
36
38
43
44

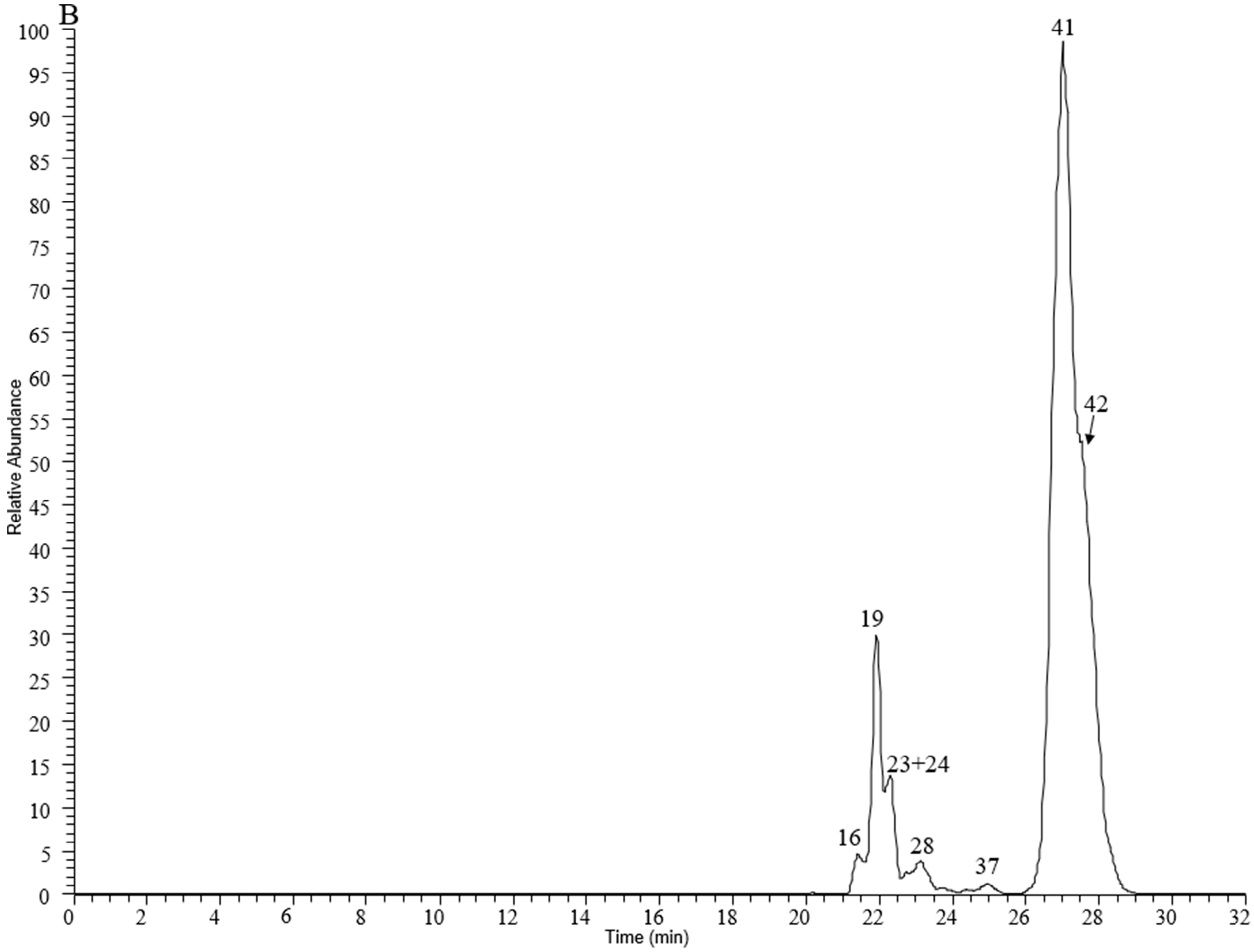

B
Relative Abundance
Time (min)
41
42
19
23+24
16
28
37

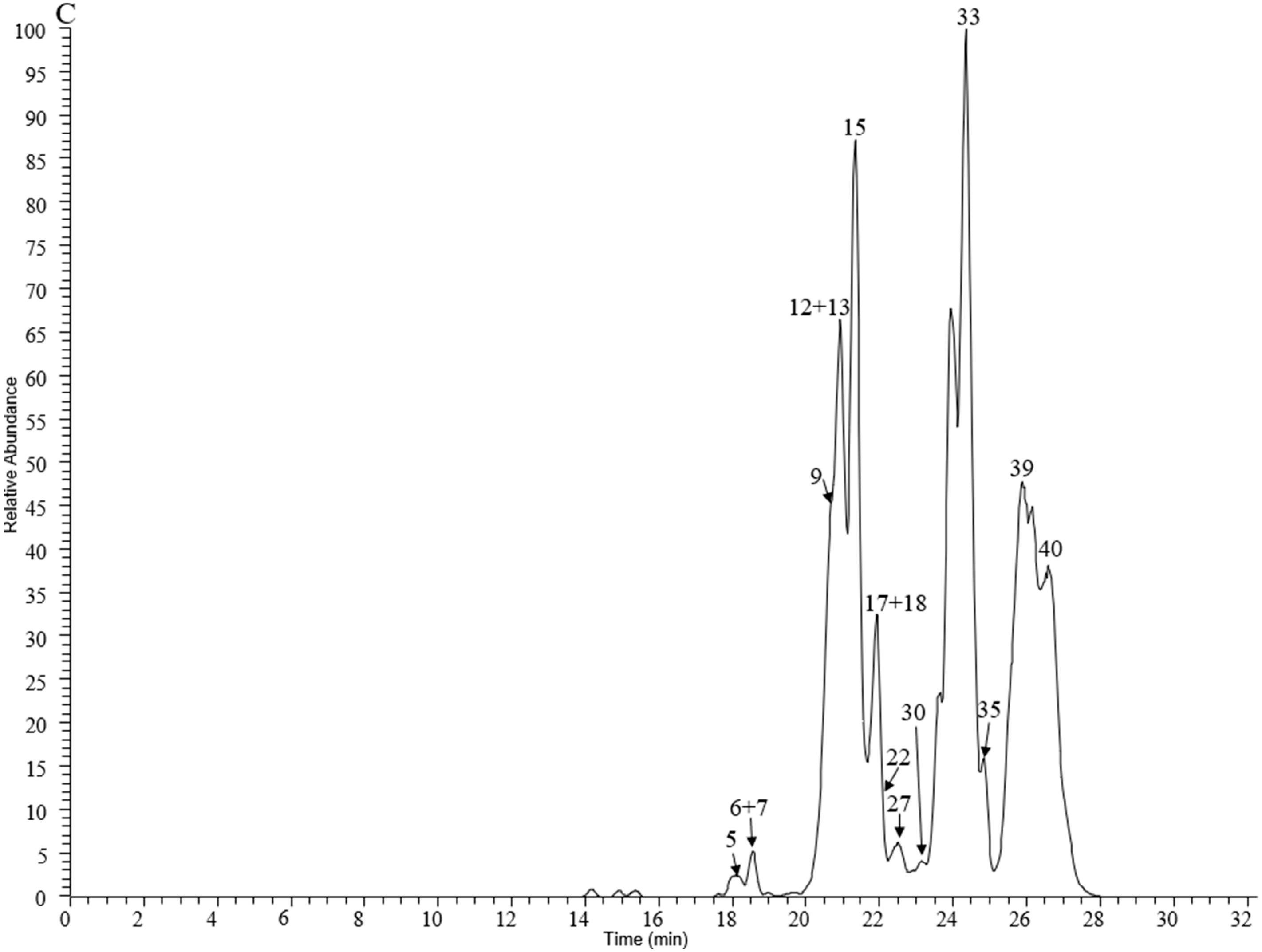
C
Relative Abundance
Time (min)
5
6+7
9
12+13
15
17+18
22
27
30
33
35
39
40

Figure S1: The high-resolution extracted ion chromatogram (HREIC) in 10 ppm for the multiple compounds in PCH. Peak 1, 3, 8, 10: *m/z* 473.1512; 2, 4, 11, 14: *m/z* 413.1301; 20, 26, 32: *m/z* 545.1723; 21, 25, 29: *m/z* 575.1828; 31, 38, 43: *m/z* 1799.5843; 34, 44: *m/z* 1961.6371; 36: *m/z* 989.3202 (**A**); Peak 16, 19, 23: *m/z* 809.2568; 24: *m/z* 1475.4787; 28, 37, 42: *m/z* 1637.5315; 41: *m/z* 1475.4787 (**B**); Peak 5, 7, 33, 35 : *m/z* 827.2674; 6, 12, 15, 17: *m/z* 503.1618; 9, 13, 39: *m/z* 1151.3730; 18, 40: *m/z* 1313.4258; 22, 27, 30: *m/z* 665.2146 (**C**).

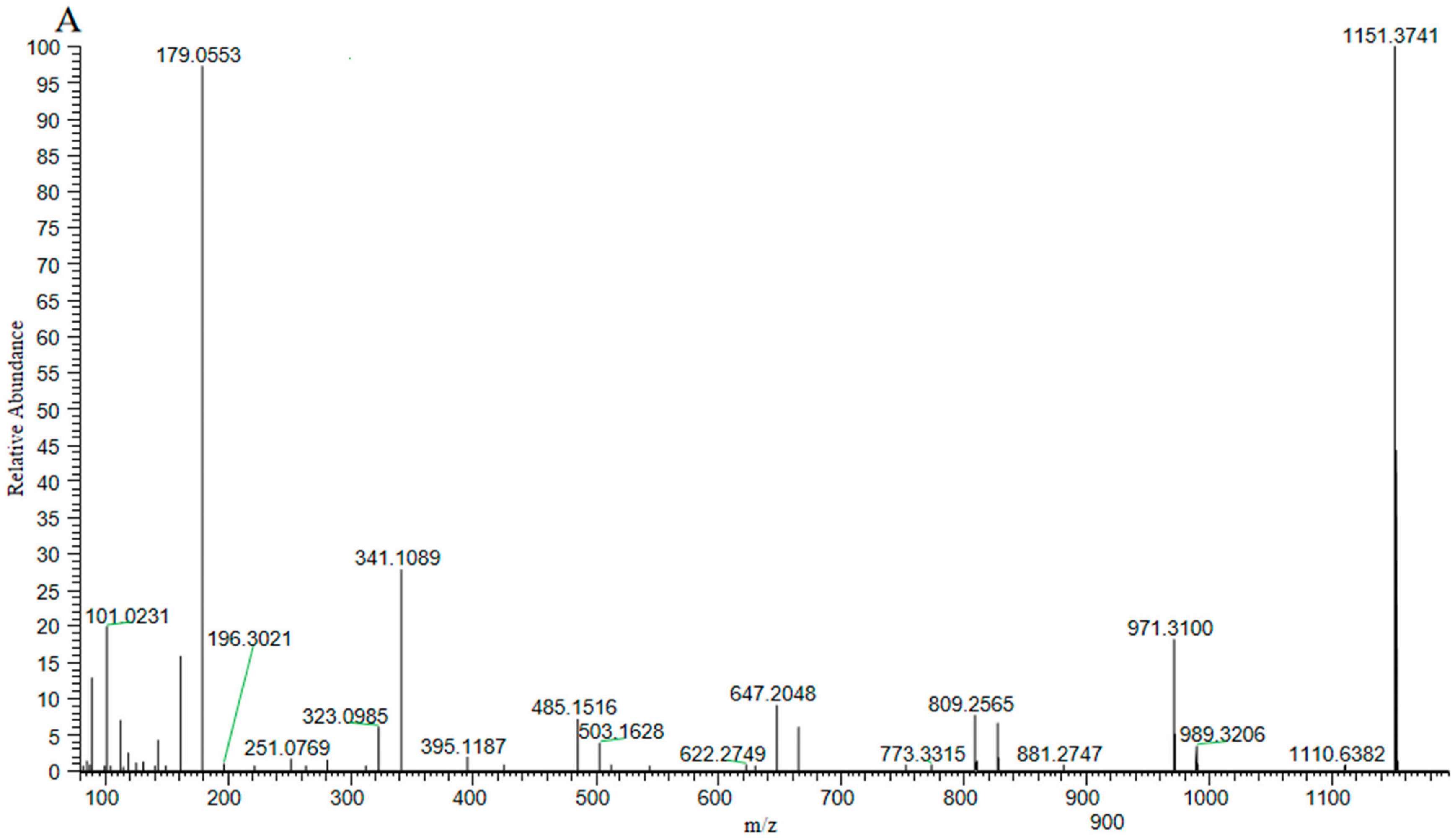
A
Relative Abundance
m/z
1151.3741
179.0553
341.1089
101.0231
196.3021
971.3100
647.2048
809.2565
485.1516
323.0985
503.1628
989.3206
251.0769
395.1187
622.2749
773.3315
881.2747
1110.6382
900

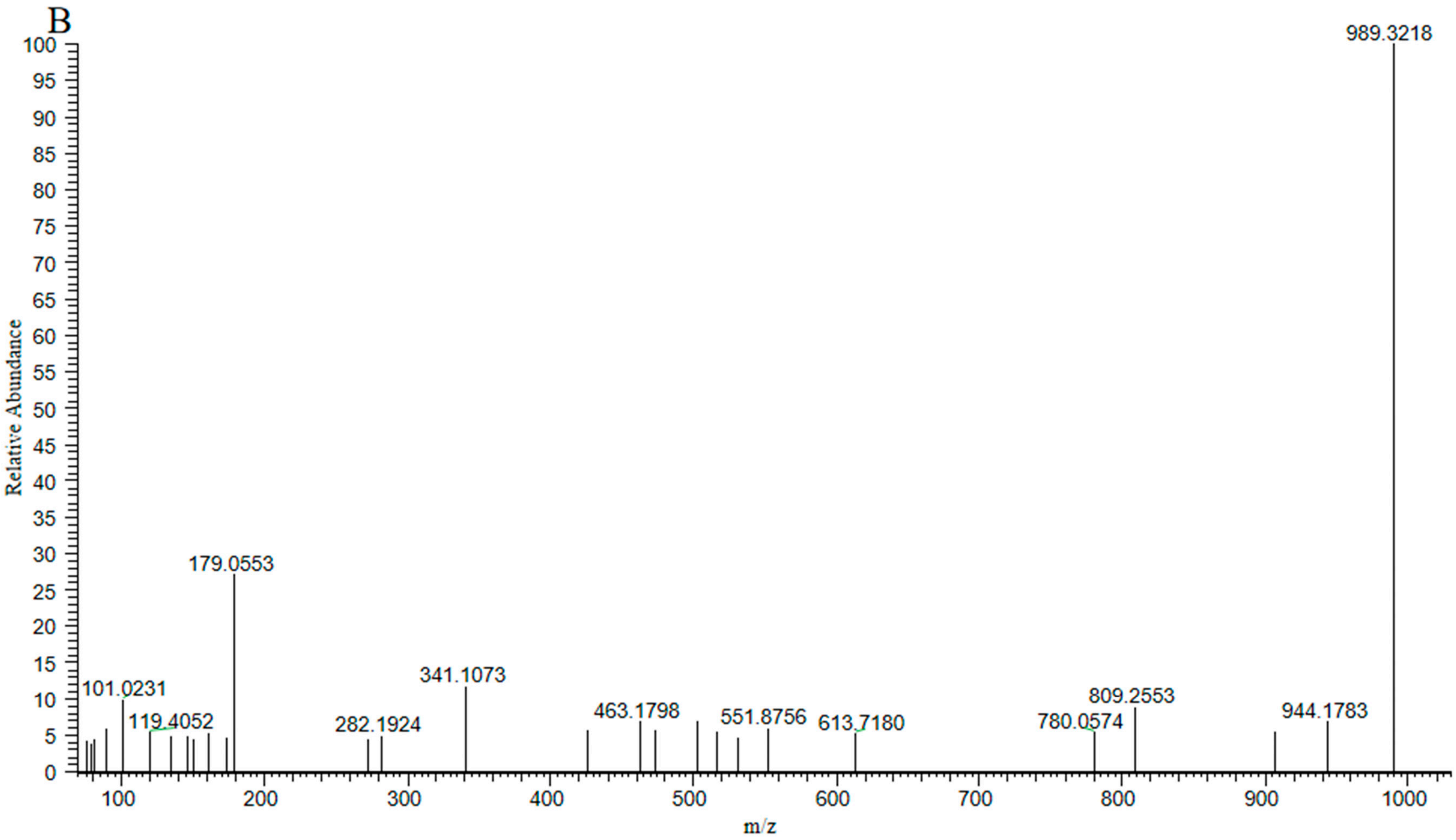
B
Relative Abundance
m/z
989.3218
179.0553
101.0231
119.4052
282.1924
341.1073
463.1798
551.8756
613.7180
780.0574
809.2553
944.1783

C

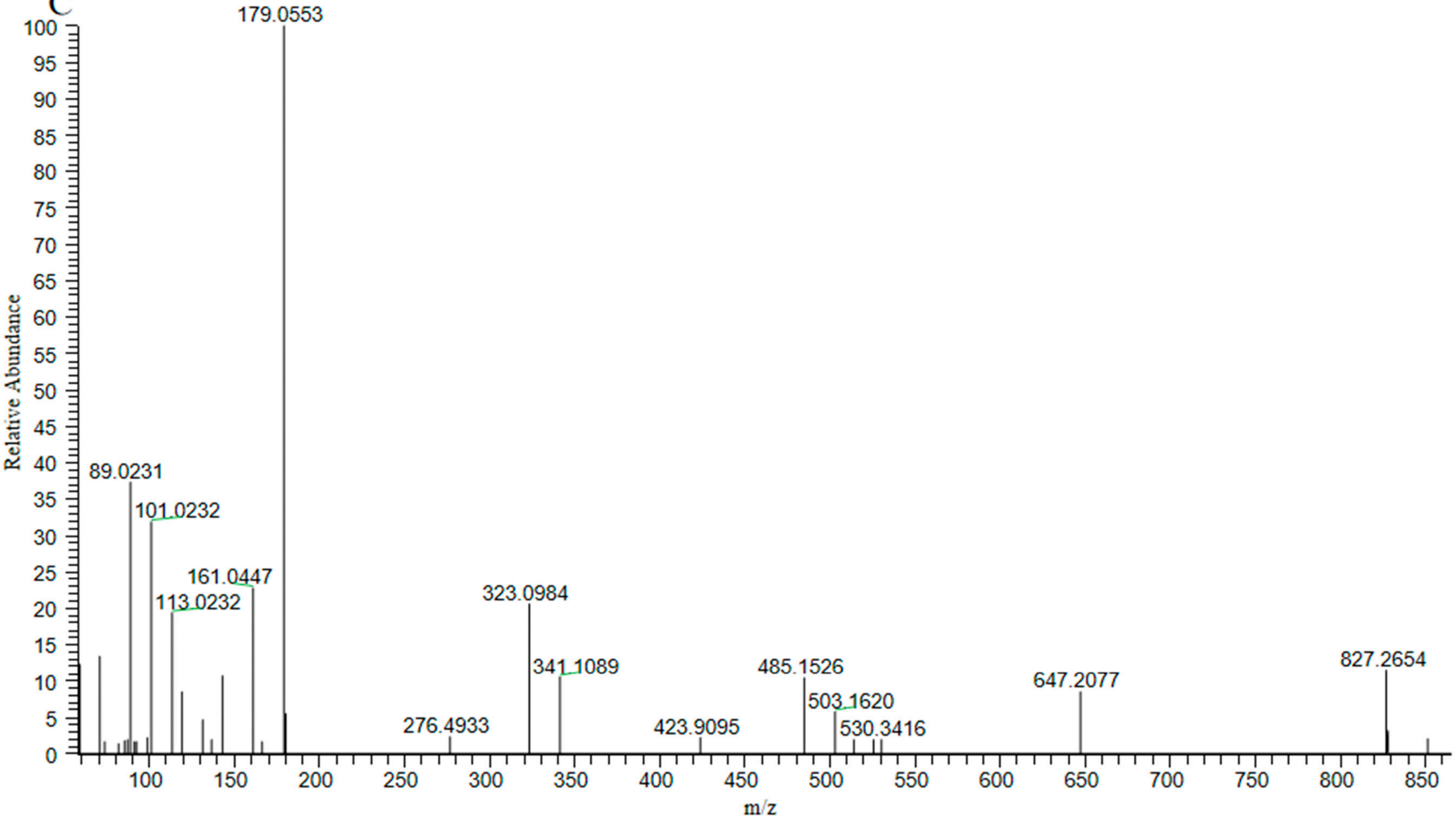
179.0553
89.0231
101.0232
161.0447
113.0232
323.0984
341.1089
485.1526
503.1620
530.3416
276.4933
423.9095
647.2077
827.2654
Relative Abundance
m/z
100
95
90
85
80
75
70
65
60
55
50
45
40
35
30
25
20
15
10
5
0
100
150
200
250
300
350
400
450
500
550
600
650
700
750
800
850

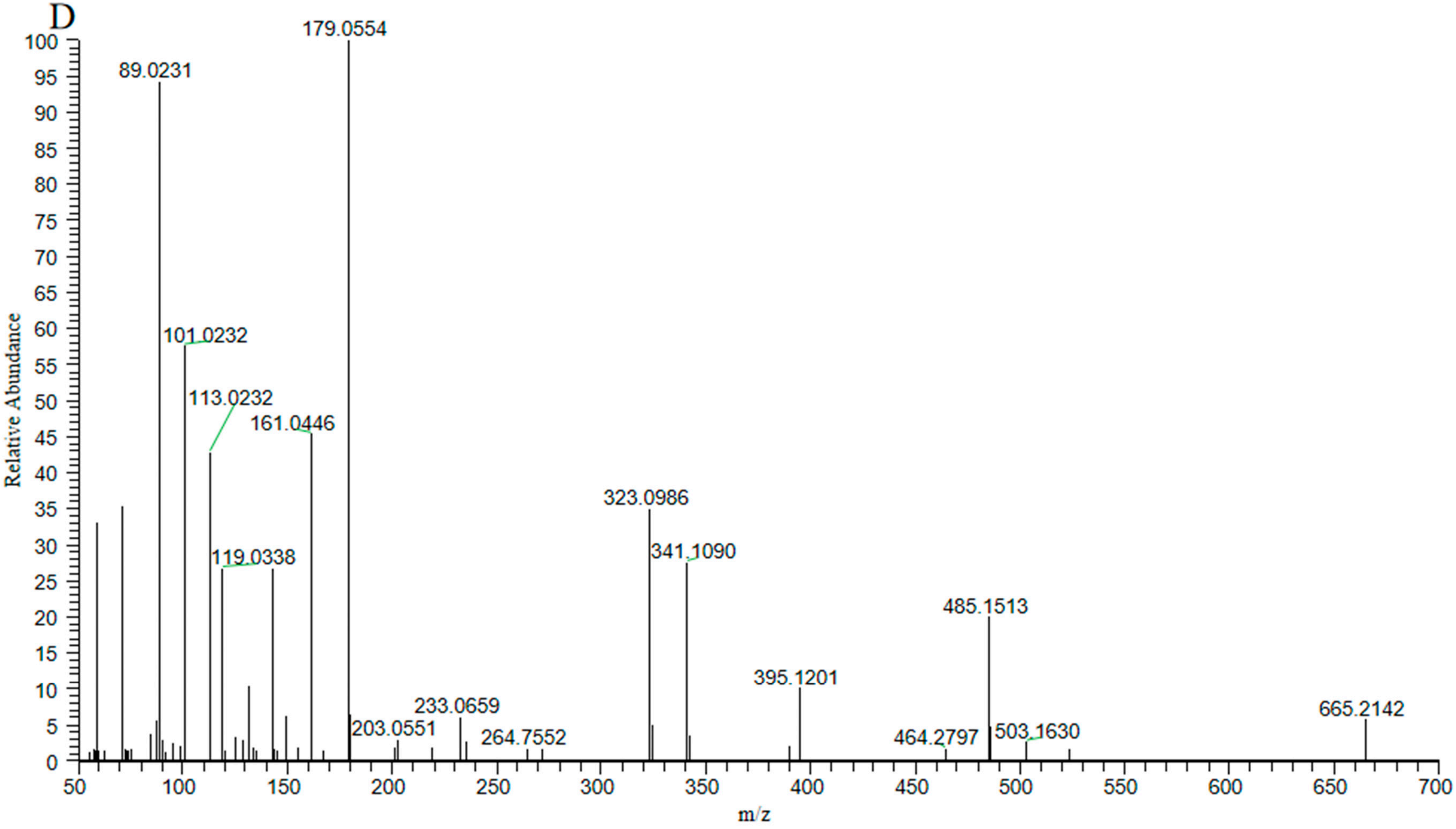
D
179.0554
89.0231
101.0232
113.0232
161.0446
119.0338
323.0986
341.1090
485.1513
395.1201
233.0659
203.0551
264.7552
464.2797
503.1630
665.2142
Relative Abundance
m/z

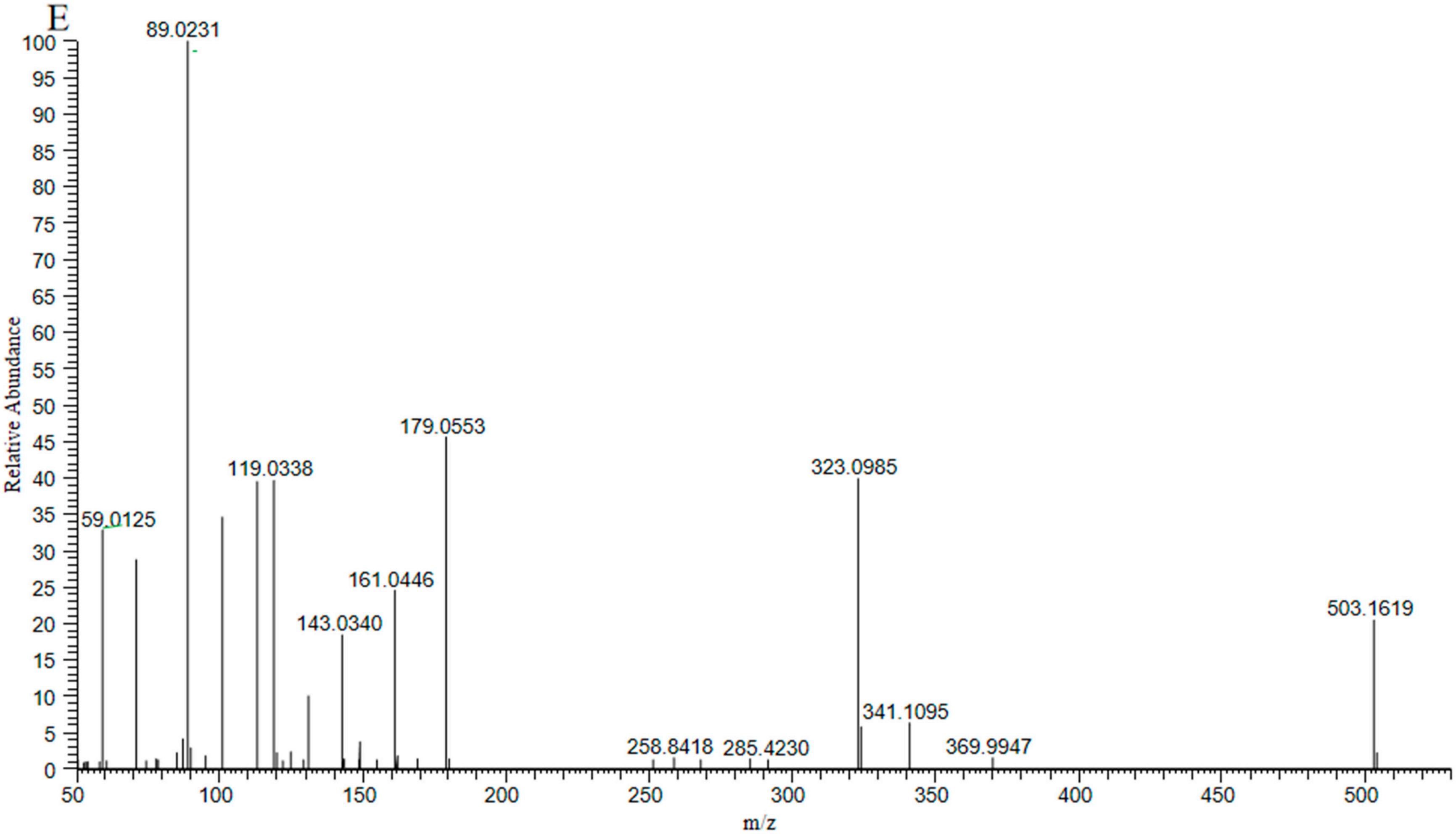

E
89.0231
179.0553
119.0338
323.0985
59.0125
161.0446
143.0340
503.1619
341.1095
258.8418
285.4230
369.9947
Relative Abundance
m/z

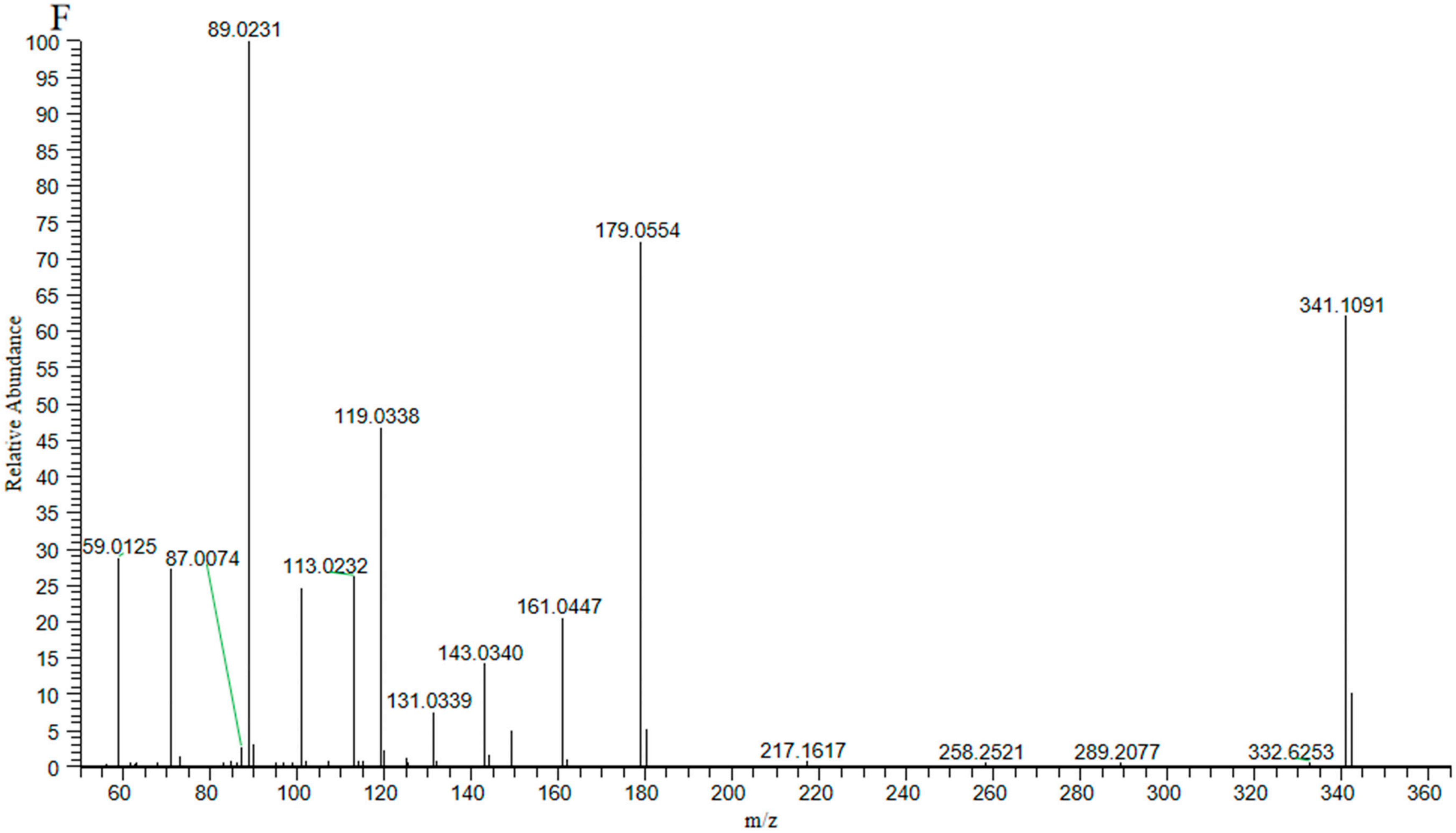
F
89.0231
179.0554
341.1091
119.0338
59.0125
87.0074
113.0232
161.0447
143.0340
131.0339
217.1617
258.2521
289.2077
332.6253
Relative Abundance
m/z

Figure S2. MS^2 spectra of the reference standards: Fructoheptasaccharide (**A**); 1,1,1,1-kestohexaose (**B**); 1F-fructofuranosyl nystose (**C**); Nystose (**D**); 1-kestose (**E**); Sucrose (**F**).
